# 297. Infectious Complications Associated with Tocilizumab Use in Patients Infected with SARS-CoV-2 at a Mid-Atlantic Hospital Consortium

**DOI:** 10.1093/ofid/ofab466.499

**Published:** 2021-12-04

**Authors:** Kristen R Kent, Nellie Darling, Xue Geng, Gavin Clark, Marybeth Kazanas, Princy N Kumar, Joseph G Timpone

**Affiliations:** 1 Georgetown University School of Medicine, Washington, District of Columbia; 2 Georgetown University, Washington, DC; 3 Georgetown University Department of Biostatistics, Bioinformatics, and Biomathematics, Washington, District of Columbia; 4 MedStar Health, Columbia, MD, Maryland; 5 MedStar Georgetown University Hospital, Washington, District of Columbia

## Abstract

**Background:**

The IL-6 inhibitor Tocilizumab (TOCI) has been associated with infections in 5-8% of patients with Rheumatoid Arthritis. TOCI has now been recommended as a treatment option for select patients with COVID-19; however, the risk of infection in this patient population is yet to be determined.

**Methods:**

We performed a retrospective chart review of patients diagnosed with COVID-19 and admitted to MedStar hospitals within the D.C./Baltimore corridor from 03/01/2020 to 12/31/2020. We identified patients who had positive culture data within 30 days of administration of TOCI-based regimens and analyzed clinical characteristics and outcomes. Univariate analyses (Wilcoxon, T-test, Chi-Square, Fisher’s Exact) were used to compare these outcome variables between patients who had post-treatment infections and those who did not.

**Results:**

A total of 220 patients received TOCI-based regimens; 16% (N=36) of patients developed positive cultures within 30 days of treatment. Of the 99 cultures, 50% were gram positive (N=49), 38% were gram negative (N=38), 10% were *Candida spp*. (N=10), and 2% were anaerobic organisms (N=2). Only 9% (8/87) of the gram positive and gram negative organisms were MDROs. Bloodstream infections were the most common and accounted for 58.4% of all infections. Length of stay (LOS) was approximately twice as long in those with post-treatment infections (26 days) compared to those without infections (14 days, p< 0.001). Although the mortality rate was higher in patients with infections after TOCI-based treatment compared to patients with no post-treatment infection (47% vs 31% respectively), this did not reach statistical significance (p=0.09). Moreover, there was no significant difference in the infection rate of patients treated with TOCI alone compared to TOCI and Dexamethasone (16.6% vs. 13.3%, p=0.99). No cases of invasive *Aspergillosis* were observed.

**Conclusion:**

Tocilizumab treatment in patients with COVID-19 may predispose patients to an increased risk of infection which is associated with a prolonged LOS and possibly higher mortality. We observed a two-fold increase in infections in COVID-19 patients compared to other patient groups receiving this treatment.

**Disclosures:**

**Princy N. Kumar, MD**, **AMGEN** (Other Financial or Material Support, Honoraria)**Eli Lilly** (Grant/Research Support)**Gilead** (Grant/Research Support, Shareholder, Other Financial or Material Support, Honoraria)**GSK** (Grant/Research Support, Shareholder, Other Financial or Material Support, Honoraria)**Merck & Co., Inc.** (Grant/Research Support, Shareholder, Other Financial or Material Support, Honoraria)

